# PcG-Mediated Higher-Order Chromatin Structures Modulate Replication Programs at the *Drosophila* BX-C

**DOI:** 10.1371/journal.pgen.1003283

**Published:** 2013-02-21

**Authors:** Federica Lo Sardo, Chiara Lanzuolo, Federico Comoglio, Marco De Bardi, Renato Paro, Valerio Orlando

**Affiliations:** 1 Dulbecco Telethon Institute, IRCCS Santa Lucia Foundation, Rome, Italy; 2 CNR Institute of Cellular Biology and Neurobiology, IRCCS Santa Lucia Foundation, Rome, Italy; 3 Department of Biosystems Science and Engineering, Swiss Federal Institute of Technology Zürich, Basel, Switzerland; 4 Neuroimmunology Unit, IRCCS Santa Lucia Foundation, Rome, Italy; 5 Faculty of Science, University of Basel, Basel, Switzerland; Max Planck Institute of Immunobiology and Epigenetics, Germany

## Abstract

Polycomb group proteins (PcG) exert conserved epigenetic functions that convey maintenance of repressed transcriptional states, via post-translational histone modifications and high order structure formation. During S-phase, in order to preserve cell identity, in addition to DNA information, PcG-chromatin-mediated epigenetic signatures need to be duplicated requiring a tight coordination between PcG proteins and replication programs. However, the interconnection between replication timing control and PcG functions remains unknown. Using *Drosophila* embryonic cell lines, we find that, while presence of specific PcG complexes and underlying transcription state are not the sole determinants of cellular replication timing, PcG-mediated higher-order structures appear to dictate the timing of replication and maintenance of the silenced state. Using published datasets we show that PRC1, PRC2, and PhoRC complexes differently correlate with replication timing of their targets. In the fully repressed BX-C, loss of function experiments revealed a synergistic role for PcG proteins in the maintenance of replication programs through the mediation of higher-order structures. Accordingly, replication timing analysis performed on two *Drosophila* cell lines differing for BX-C gene expression states, PcG distribution, and chromatin domain conformation revealed a cell-type-specific replication program that mirrors lineage-specific BX-C higher-order structures. Our work suggests that PcG complexes, by regulating higher-order chromatin structure at their target sites, contribute to the definition and the maintenance of genomic structural domains where genes showing the same epigenetic state replicate at the same time.

## Author Summary

DNA replication is a tightly orchestrated process that precisely duplicates the entire genome during cell division to ensure that daughter cells inherit the same genetic information. The genome is replicated following a specific temporal program, where different segments replicate in distinct moments of the S phase correlating with active (early) and repressed (late) transcriptional state of resident genes. Moreover, replicating chromosomal domains are organized in the nuclear space, perhaps to guarantee the conservation of the same topological order in daughter cells. Epigenetic mechanisms, acting via chromatin organization, determine transcriptional states and must be maintained through cell division. Here, we analyzed in detail the link between Polycomb Group (PcG) proteins, higher-order chromatin structure, and replication timing in *Drosophila*. By using bioinformatic analyses combined with functional experiments, we show that Polycomb Repressive Complex 1 (PRC1), PRC2, and PhoRC differently correlate with replication timing of their targets and that transcription *per se* does not determine replication timing. Strikingly, by analyzing the PcG-regulated Bithorax Complex, where PRC1, PRC2, and PhoRC complexes are bound to repressed targets, we provide evidence for a synergistic role of PcG proteins in the modulation and maintenance of replication timing through the definition of specific, topologically distinct genomic domains.

## Introduction

One of the key open questions in biology is how epigenetic traits are faithfully duplicated during the cell cycle and how this safeguards the correct maintenance of transcriptional programs and cell identity. During S-phase, replication of chromatin domains containing differentially expressed genes appears to be regulated in a spatial and temporal manner. In general it is widely accepted that active transcriptional units are preferentially replicated early whereas silenced genes and heterochromatin are replicated in late S-phase [1]. However, the contribution of epigenetic regulators to this dynamics remains to be elucidated.

Polycomb group (PcG) multiprotein complexes are evolutionary conserved epigenetic regulators required for the maintenance of repressed transcriptional states during development and in adult tissues [2]. In *Drosophila melanogaster* five PcG complexes have been identified, controlling gene silencing at different levels by regulating RNA Pol II function, histone modifications and higher-order chromatin structures; Polycomb repressive complexes 1 (PRC1) and 2 (PRC2), Pho-repressive complex (PhoRC), dRing-associated factors (dRAF) complex and Polycomb repressive deubiquitinase (PR-DUB) complex [2]. PcG complexes exert their function by interacting with specialized cis-regulatory regions termed PcG Response Elements (PREs) [3], [4] and with transcription start sites (TSSs) [5]. The zinc finger protein Pleiohometic (PHO) of PhoRC is thought to play an important role in PRC1 and PRC2 recruitment [6]. Once recruited, the PRC2 complex, via its catalytic subunit E(z), deposits the characteristic repressive chromatin mark, histone H3 trimethylated at lysine 27 (H3K27me3) [7]–[9], which in turn serves as docking site for PRC1 [10]. Previous works have revealed that PcG-bound regulatory regions can interact with promoters and modulate their activity via mechanisms involving looping between regulatory elements and long-distance interactions in *cis* or in *trans* (between different chromosomes) [11]–[13]. The genome is topologically organized into chromatin loops also during the process of DNA replication, when hundreds of replication factories are formed, each containing clusters of replication origins that fire almost simultaneously [14]. It has been proposed that, in these replication foci, neighbouring origins are located in physical proximity to each other while inter-origin DNA regions are looped out, forming rosette-like structures [15], [16]. In each factory large segments of the chromosome are replicated in a coordinated manner at characteristic times during S-phase. In higher eukaryotes the choice of replication origins and the time of firing are cell-type specific [17]–[19] and change dynamically during differentiation and development [20]–[22]. It has been proposed that epigenetic mechanisms regulating chromatin structure and chromosome organization could play a role in regulating the selection of DNA replication origins and the time of firing [23]–[26]. Conversely, timing and selection of replication origins can contribute to the establishment of chromatin domains thus modulating transcription [27].

In *Drosophila* it was shown that at the 340 kb, PcG-repressed homeotic Bithorax Complex (BX-C), distally spaced PREs, promoters and 3′ ends of repressed genes interact with each other to form multi-looped structures that are dynamically regulated during replication [13], [28]. In order to investigate the functional role of PcG-mediated silencing and higher-order chromatin structures in the modulation of replication programs in *Drosophila*, we used cell lines derived from embryonic tissue as model system. By means of genome-wide bioinformatic and statistical analyses we investigated whether PRC1, PRC2 or PhoRC bound regions would show any preferential replication timing in *Drosophila* embryonic Schneider 2 cell line (S2). We found that PRC2-targeted promoters replicate later than non-target ones, in agreement with the generally accepted concept that inactive genes are, on average, late replicating [28]–[33]. However, no significant difference in replication timing distributions emerged between PRC1 or Pho-RC bound and non-bound promoters. To gain deeper insight on the interplay between PcG-mediated silencing, higher-order structures and replication timing we focused on the repressed and late replicating BX-C in S2 cells. We found that, simple derepression of BX-C genes does not result in changes in replication timing or higher-order structures, while a combined PcG dependent (PHO, PC and E(z)) impairment of BX-C higher-order structures is accompanied by an anticipation in replication timing. Further, we found that epigenetically distinct cell lines, differing in BX-C gene transcription and topological three-dimensional conformations show PRE specific replication timing profiles. Our work reveals that PcG mediated BX-C higher-order structures coincide with replication domains and that PcG complexes act synergistically for the epigenetic maintenance of replication timing programs.

## Results

### PRC1-, PRC2-, and PhoRC-bound promoters show different replication timing distributions in *Drosophila* S2 cells

A positive correlation between PcG-mediated H3K27me3 mark and late replication has been previously reported [34]. Using public available data sets we analyzed the genome-wide replication timing distribution of PRC2, PhoRC and PRC1 bound promoters, defined as all regions within 500 bp of a unique RefSeq Transcription Start site (TSS). The replication timing of each promoter was estimated as described in Material and Methods. As representatives of PRC2 complex and its enzymatic activity we used, respectively, E(z) and H3K27me3 whereas PHO was used as representative of the PhoRC complex. All three datasets are available from modENCODE as ChIP-chip genome-wide profiles (see Material and Methods for details). Feature enrichments at promoters and their statistical significance have been computed using a conservative approach as described in Material and Methods.

**Figure 1:**
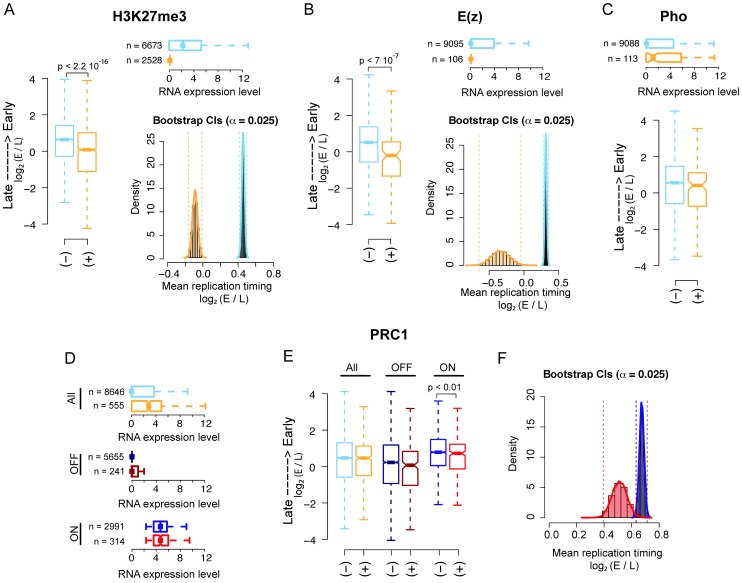
PRC2, PhoRC, and PRC1 show different replication timing distributions in *Drosophila* S2 cells. (A) Boxplot (left panel) of genome-wide *Drosophila* embryonic S2 cell line replication timing for significantly H3K27me3 enriched promoters (orange) and non-enriched promoters (light blue). Horizontal boxplot (top right panel) of RNA expression levels in the two previous groups, where group sizes are indicated to the left. Bootstrap distributions (bottom right panel) of mean replication timing for H3K27me3 significantly enriched promoters (orange) and non-enriched promoters (light blue). Percentile confidence intervals (α = 0.025) are indicated with dashed vertical lines. (B) Same as (A), with promoters classified as E(z) bound (orange) and non-bound (light blue). (C) Same as the first two panels of (B), with promoters classified as PHO bound (orange) and non-bound (light blue). (D) Horizontal boxplot of RNA expression levels for genome-wide PRC1 bound (orange) and non-bound (light blue) promoters, PRC1 bound (dark red) and non-bound (dark blue) OFF promoters only and PRC1 bound (red) and non-bound (blue) ON promoters only (see Material and Methods). Group sizes are indicated to the left. (E) Boxplot of replication timing for the three previous groups. (F) Bootstrap distributions of mean replication timing for PRC1 bound (red) and non-bound (blue) ON promoters. Percentile confidence intervals (α = 0.025) are indicated with dashed vertical lines. All p-values have been computed using a Wilcoxon rank sum test.

First, we found that both H3K27me3 and E(z) enriched regions replicate significantly later than their non-enriched counterparts as shown by both boxplot and percentile bootstrap confidence intervals, left and right panel, respectively in Figure 1A and 1B. Of note, 94% of E(z) bound promoters are also significantly enriched for H3K27me3 in our analysis. To test whether the observed difference in replication timing could be entirely explained by the significantly lower transcriptional activity of PRC2 bound promoters (Figure 1A, 1B, upper panels), we used weighted bootstrap to compute confidence intervals for the mean replication timing of H3K27me3 enriched and non-enriched promoters using probability weights that account for the different transcriptional activity distributions between the two groups (see Materials and Methods for details).

Interestingly, the distributions of the mean replication timing within the two groups remained largely separated after resampling (Figure S1A and Materials and Methods), suggesting that transcription might not be the sole determinant of late replication timing for H3K27me3 enriched promoters.

Second, we analyzed the correlation between PHO binding and replication timing and we did not find any significant difference in the mean replication timing of PHO bound promoters compared to non-bound promoters as shown in Figure 1C, lower panel. The upper panel shows the transcriptional activity of the promoters in the two groups.

Third, we investigated whether PRC1 binding at promoters significantly correlates with their replication timing. We defined PRC1 bound those promoter regions showing joint enrichment for all three PRC1 core components: PC, Ph and Psc. All three proteins were profiled genome-wide in [5] using ChIP-Seq. Differently from what we observed for H3K27me3 and E(z) bound promoters, PRC1 bound promoters are globally more transcribed than their non-bound counterparts (Figure 1D, upper panel). When all promoters were considered, we did not find any significant difference between the mean replication timing of PRC1 bound and non-bound promoters (Figure 1E, left boxplot). The differential replication timing distribution between PRC1, PhoRC and PRC2 may reflect a specific regulatory difference between these complexes, which has already been reported in previous works [5], [35]–[37]. Moreover, these data support emerging evidence that PRC bound promoters are not universally silent [5], [37]–[40]. PRC1 components have been shown to colocalize with TrxG complexes on stalled promoters, where RNA Pol II is “poised” for subsequent activation in response to developmental cues [5]. These promoters could be functionally considered as the fly's analogs of the ‘bivalent domains’ found in mammals, representing poised states for lineage-specific activation of key regulatory and developmental genes [36], [39]. To quantify the contribution of bivalent PRC1 bound regions to replication timing, we compared all promoters bound by PRC1 with promoters co-bound by PRC1 and Trx (Figure S1B), finding no difference between the two classes. To dissect the link between transcription and replication timing of PRC1 bound promoters, we divided them into two subgroups: OFF promoters, represented by promoters with none or little transcriptional output, and ON promoters containing actively transcribed promoters (Figure 1D, mid and lower panel, respectively, see Material and Methods). Of note, approximately half of the stalled promoters were considered ON in our analysis according to this definition. We then analyzed the mean replication timing within each subgroup. Interestingly, although OFF promoters are late replicating, no significant difference in replication timing distribution was found between PRC1 bound and non-bound (Figure 1E, mid boxplot). This result might be explained by the considerable amount of late replicating constitutive heterochromatic promoters that are not occupied by PRC1, thus masking a possible contribution of PRC1 to replication program regulation. On the other hand, PRC1 bound ON promoters are significantly later replicating than PRC1 non-bound ON promoters (Figure 1E, right boxplot) despite the two sets exhibit no significantly different transcriptional activity (Figure 1D, lower boxplot). This result is in agreement with recent observations showing active transcription of PRC1 bound promoters [5] and reinforces the idea that transcription is not the unique discriminator of replication timing of a given locus. Figure 1F shows percentile bootstrap confidence intervals for the mean replication timing within the two groups of active promoters.

Taken together, these results corroborate the view of a functional difference between PRC2, PhoRC and PRC1 complexes and suggest that PRC2 complex, but not PRC1 or PhoRC, can be considered a genome-wide predictor of replication timing in *Drosophila*.

### Forced transcriptional reactivation by depletion of single PcG complex subunits changes neither replication timing nor the overall BX-C higher-order interactions

**Figure 2:**
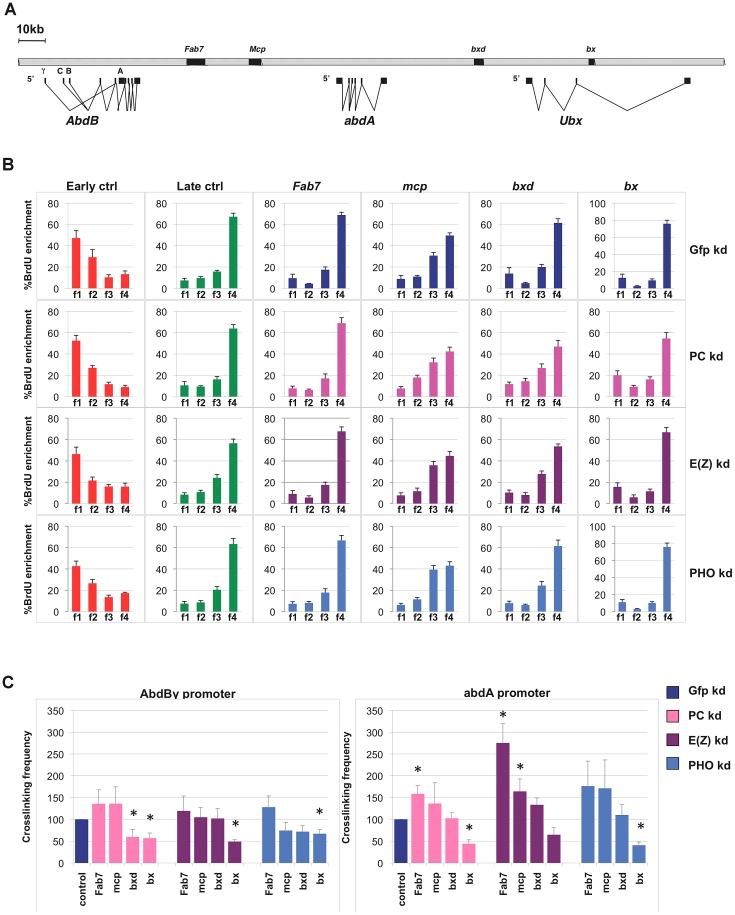
Single PcG subunit depletion does not affect BX-C replication timing or higher-order structures. (A) Schematic representation of the Bithorax Complex (BX-C) including homeotic genes and characterized PREs. (B) Enrichment of BrdU labelled DNA in the four FACS sorted S-phase fractions as quantified by real time PCR with indicated primers in control cells (blue) and in cells treated with PC-dsRNA, E(z)-dsRNA or PHO-dsRNA (pink, purple and light blue, respectively). The relative abundance of locus-specific DNA in each cell-cycle fraction is calculated from the average values of threshold cycle (Ct), normalized to the Ct of a mitochondrial sequence as internal control (Ctmit), using the following equation: 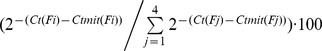, where *i* is one of the four fractions. All data points were generated from an average of six independent experiments. Standard error of the mean is indicated. (C) Crosslinking frequencies, normalized to the internal control, between the fixed fragments spanning two homeotic promoters (*AbdBγ* and *abdA*) and BX-C PREs. Crosslinking frequencies observed in GFP–dsRNA-treated cells are shown in blue while data obtained in cells treated with PC-dsRNA E(z)-dsRNA or PHO-dsRNA are in pink, purple and light blue, respectively. Standard error of the mean is indicated. All data points were generated from an average of four independent biological replicates. Two-tailed t-test was applied for statistical analysis. Asterisks indicate statistically relevant differences: (α = 0.05). P values: PC-dsRNA treated cells: *AbdBγp/bxd*: P = 0,04 *AbdBγp/bx*: P = 0,03×10^−1^; *abdA/Fab7*: P = 0,01; *abdA/bx*: P = 0,01×10^−2^; E(z)-dsRNA treated cells: *AbdBγp/bx*: P = 0,06×10^−6^; *abdA/Fab7* P = 0,07×10^−2^; *abdA/mcp*: P = 0,04; PHO-dsRNA treated cells *AbdBγp/bx*: P = 0,02×10^−1^; *abdA/bx*: P = 0,09×10^−4^.

To further investigate the possible contribution of PRC1 and other PcG complexes to replication timing at repressed targets (OFF TSSs), we focused our attention on the fully silenced and late replicating locus BX-C (Figure 2A). We performed loss of function experiments by treating *Drosophila* S2 cells with dsRNA against mRNA encoding either PHO, E(z) or PC proteins, members of the PhoRC, PRC2 and PRC1 complexes respectively, in order to evaluate and dissect the contribution of PcG complexes on BX-C transcriptional silencing, higher-order structures maintenance and replication program control. Extending previous reports [13], after single PcG-knockdown we observed a transcriptional reactivation of all three BX-C homeotic genes accompanied by a mild derepression of the intervening non-coding transcripts encompassing PREs (Figure S2A). Notably, depletion of specific PcG proteins differentially affects BX-C transcription. In particular PC depletion results in a stronger derepression, causing an increase of transcription up to ten thousand fold for the *Ubx* transcript. In contrast, E(z) or PHO depletions, despite their suggested role in PRC1 recruitment [6] cause a weaker transcriptional increase. Massive transcription in the region corresponding to *bx* PRE (Figure S2A, right panel) in PC depleted cells reflects the strong activation of *Ubx* homeotic gene transcript encompassing *bx* region (Figure S2A, left panel). The different transcriptional effects observed upon depletion of each specific PcG subunit did not depend on interference efficiency, as all targeted proteins were not detected by western blot after three rounds of dsRNA treatment (Figure S2B).

To determine BX-C replication timing, we pulse-labelled asynchronous S2 cells with BrdU and FACS sorted four S-phase fractions, from the earliest (f1) to the latest (f4), according to DNA content (Figure S2C). DNA was prepared from an equal number of cells representing the four fractions. BrdU-labelled DNA was immunoprecipitated from these fractions to enrich for those genomic sequences that replicate during the labelling period. We then performed quantitative real-time PCR (qRT-PCR), using primers specific for control sequence regions previously shown in S2 cells to be early and late replicating [34], for *Fab-7*, *Mcp*, *bxd* and *bx* PREs and for homeotic gene promoters (Figure 2B, Figure S2D). As an internal control we used a unique region of the mitochondrial genome, expected to replicate throughout the cell cycle (see Materials and Methods for details). Our analysis showed that repressed BX-C PREs replicate during late S phase in control cells, being enriched in the latest S-phase fraction (Figure 2B). In PcG-depleted cells where PREs and homeotic genes were no longer repressed, late replication was maintained (Figure 2B, Figure S2D), suggesting that single PcG proteins do not have a strong influence on BX-C replication programs. This result was confirmed by statistical analysis, in which we calculated the ratio between the amounts of amplified products in the earliest (f1) and the latest (f4) S-phase fraction (Figure S2E). Interestingly, *abdA* and *Ubx* promoters were late replicating in control cells, while the 5′ region of the *AbdB* gene, being situated in a transition region of replication timing (Figure S3), shows an intermediate timing of replication, presenting the highest BrdU incorporation in f2 and f3 S-phase fractions (Figure S2D). We have previously shown that reduced levels of the single PRC1 subunit Polycomb (PC) determines minor changes in higher-order structures [13]. Similar results were obtained in mammals [41], [42]. In order to investigate to what extent BX-C higher-order structures are affected by depletion of different PcG proteins, we used Chromosome Conformation Capture (3C) analysis to monitor DNA/DNA interactions between PcG targets. Comparison of crosslinking frequencies in depleted versus control cells reveals that PRE/promoter interactions were affected more in PC depleted than in E(z) and PHO depleted cells, but the overall BX-C structure was maintained upon single PcG knock-down (Figure 2C), reinforcing and extending previous reports [13]. Notably, E(z) depletion caused an increased frequency of some interactions between *abdA* promoter and PREs (Figure 2C, right panel). Taken together, these data indicate that transcriptional reactivation of homeotic genes after depletion of single PcG subunits, only partially impairs BX-C three-dimensional structure and it is not sufficient *per se* to change the replication timing.

### Simultaneous depletion of multiple subunits of PcG protein complexes determines changes of BX-C transcription, replication timing, and high-order structures

**Figure 3:**
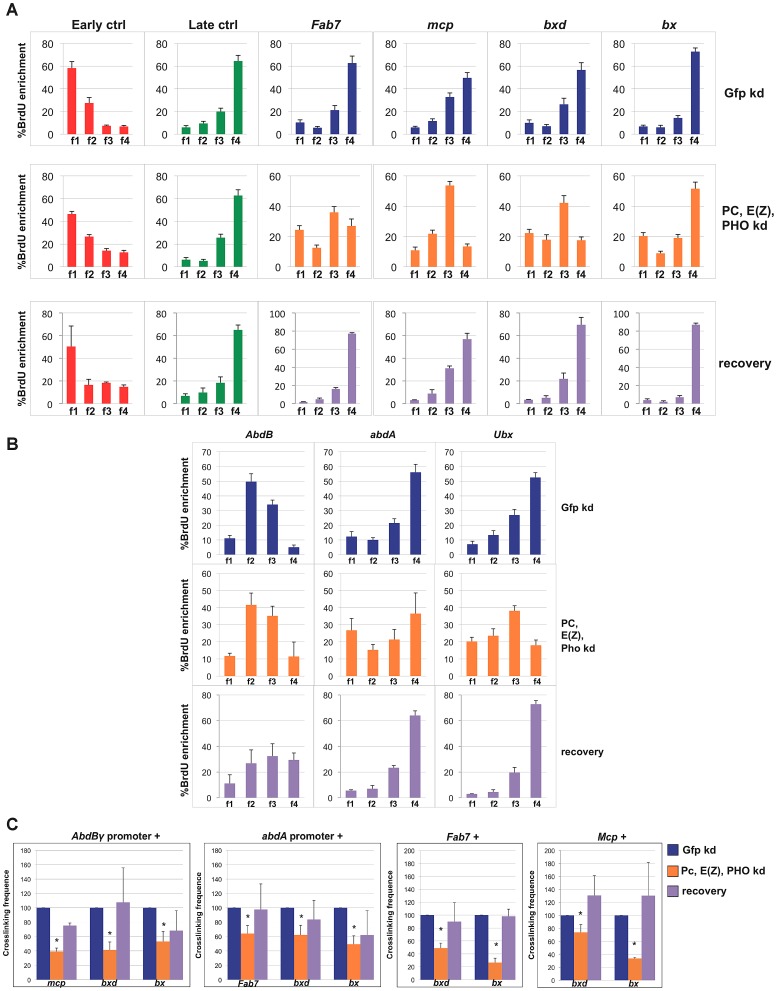
Simultaneous depletion of three PcG proteins determines reversible changes of BX-C higher-order interactions and replication timing. Data obtained in control cells are shown in blue, data obtained in cells treated with dsRNA against PC, E(z) and PHO and in recovered cells are in orange and violet, respectively. (A, B) Enrichment of BrdU labelled DNA in the four FACS sorted S-phase fractions as quantified by real time PCR with indicated primers of controls and PREs (A) or homeotic genes promoters (B) as measured by quantitative RT-PCR. All data points were generated from an average of at least four independent experiments. Standard error of the mean is indicated. (C) Left panels: crosslinking frequencies, normalized to the control, between the fixed fragments spanning two homeotic promoters (*AbdBγ* and *ab*dA) and BX-C PREs in triple PcG depleted cells and recovered cells; right panels: crosslinking frequencies, normalized to the control, between the indicated PREs. Asterisks indicate statistically relevant differences. Standard error of the mean is indicated. Two-tailed t-test was applied for statistical analysis. α = 0.05. P values: *AbdBγp/mcp* P 0,05×10^−7^; *AbdBp/bxd* P = 0,01×10^−1^; *AbdBγp*/*bx* P = 0,02; *abdAp/fab7* P = 0,01×10^−2^. *abdAp*/*bxd* P = 0,02; *abdAp/bx* P = 0,04×10^−7^; *Fab-7/bxd* P = 0,07×10^−2^; *Fab-7/bx* P = 0,02×10^−5^. *mcp/bxd* P = 0,02; *mcp/bx* P = 0,07×10^−9^.

We went on and performed simultaneous depletions of the PHO, E(z) and PC subunits. As shown in Figure S4A and S4B, mRNA and protein levels of targeted PcG subunits were consistently reduced. As expected, in triple PcG depleted cells we observed a transcriptional reactivation of homeotic genes one order of magnitude higher than in a single PcG knock-down (Figure S4C). In addition, we found that transcription through PREs quantitatively correlates with homeotic gene reactivation (Figure S4D) being nearly ten fold for *Fab-7*, *Mcp* and *bxd* in respect to the GFP control. Strikingly, when we performed replication timing analyses in triple depleted cells, a clear anticipation of *Fab7*, *Mcp* and *bxd* replication timing was observed, being enriched in f3 S-phase fraction after PcG depletion (Figure 3A). Analysis done on two S phase fractions (Figure S4E) confirmed the above changes and identified the indicated PREs as mid replicating sequences, becoming statistically different from both early and late replicating sequences. Interestingly, each PRE was differently affected, with *bx* not susceptible to the triple PcG protein depletion (Figure 3A, Figure S4E). Notably, this trend does not correlate with a higher degree of transcriptional reactivation (Figure S4D), suggesting again that transcription *per se* does not influence replication timing. We then measured replication timing of homeotic gene promoters and we found that only *Ubx* gene promoter showed anticipation in replication timing after triple PcG depletion (Figure 3B, Figure S4E).

We further analysed functional DNA/DNA interactions in triple PcG depleted cells. Interestingly, 3C analysis revealed that, although homeotic gene promoters (*AbdB*γ promoter and *abdA* promoter) maintained the association with their functional PRE (*Fab-7* and *Mcp*, respectively, Figure S4F), other promoter/PRE and PRE/PRE interactions were impaired (Figure 3C). Of note, the overall BX-C conformation was not completely lost (Figure S4F), suggesting the presence of other regulators of BX-C structure that are not affected by PcG depletion.

In order to follow the stability of the loss of function phenotype, PcG depleted, BX-C reactivated cells were grown for additional 30 days (almost 20 cell divisions) in the absence of dsRNA to restore normal PcG levels. In all the independent samples we found a complete recovery of PcG transcripts and a partial recovery of protein levels (Figure S4A and S4B). In recovered cells, we found a general tendency to restore BX-C repression, here partially re-established for *Ubx* and *abdA* and fully for *AbdB* (Figure S4C), in agreement with previous findings in imaginal discs [43]. Similarly, PRE transcripts were re-repressed with the exception of *bx* that reflects the higher level of derepression of *Ubx* (Figure S4D). To exclude that the recovery effect could be due to poorly depleted cells that could have repopulated the culture, we compared the proliferation of control cells with triple depleted cells after each round of transfection (see Material and Methods). The results showed no difference between the proliferative potential of both type of cells (Figure S4G). We then measured PRE replication timing and crosslinking frequency between PREs and promoters in recovered cells. After 30 days BX-C late replication timing was restored in recovered cells, showing values indistinguishable from control cells (Figure 3A, 3B; Figure S4E, S4H). Concomitantly, we observed a complete recovery of PRE/promoter and PRE/PRE interactions (Figure 3C). These data indicate that differences in replication timing and higher-order structures were influenced by a temporary loss of multiple PcG proteins. This effect can be reversed when wild type conditions are re-established, thus indicating that PcG complexes act synergistically to maintain programmed silencing, topological order and replication timing at BX-C.

### A different topological order at the BX-C correlates with distinct replication timing programs

**Figure 4:**
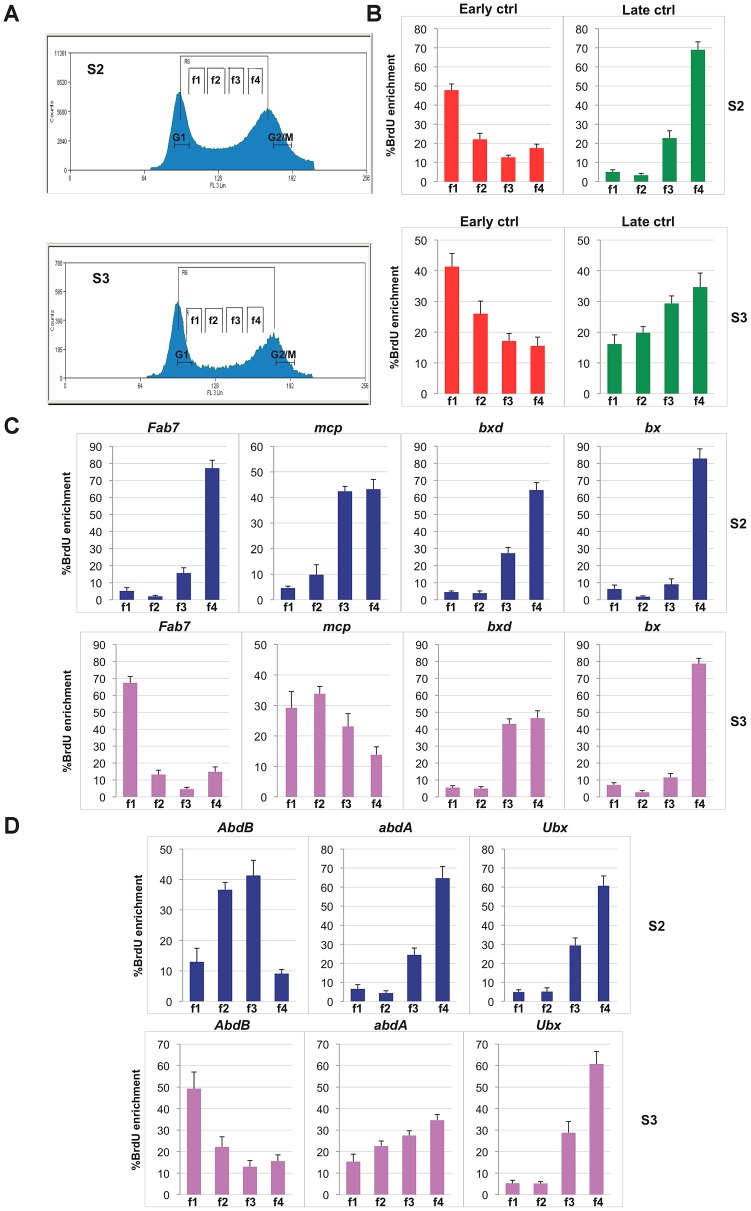
Different replication timing profiles at BX-C PREs in S2 and S3 cell lines. (A) Cell-cycle profiles of *D. melanogaster* S2 cells (top) and S3 cells (bottom) after BrdU pulse labelling and propidium iodide staining. Cells between the G1 and G2 peaks are in S phase. Gates indicate the sorted fractions: f1 represents the earliest and f4 the latest S-phase fraction. (B, C, D) Enrichment of BrdU labelled DNA in the four FACS sorted fractions as quantified by real time PCR with primers specific for early and late replication timing controls (B), PREs (C) and homeotic gene promoters (D) in S2 and S3 cell lines. All data points were generated from an average of at least four independent experiments. Standard error of the mean is indicated.

To understand whether early established PcG-mediated chromatin structures are associated with specific replication programs, we analysed two *Drosophila* embryonic cell lines, S2 and S3, showing distinct BX-C gene expression and structural conformation [13]. We have previously shown that in S2 as in S3 cells, *Ubx* and *abdA* are repressed or transcribed at low levels, while all three *AbdB* and the downstream intervening non-coding transcripts are strongly expressed only in S3 cells [13], [44] (Figure S5A). In these cells the *AbdB* domain features several epigenome structural differences in respect to S2 cells, such as reduced PcG protein binding [38], different histone mark enrichment [44] and different higher-order chromatin interactions [13]. While in S2 cells all PcG binding sites are clustered and mediate the formation of a multi-loop higher-order structure, in S3 cells the genomic section containing the *AbdB* gene as well as the *Fab-7* and *Mcp* PREs loses contact with other repressed PcG bound elements of the BX-C cluster, creating a distinct domain [13] (Figure S5B). We performed replication timing analysis in S3 in comparison to S2 cells (Figure 4A). In S3 cell line, we observed a different replication timing profile for specific BX-C PREs (Figure 4B and 4C). In particular, while in S2 cells all PREs are enriched in the f4 fraction, representing the latest S phase (Figure 4C and S5C), in S3 cells, repressed *bx* and *bxd* PREs are enriched in the late S-phase fraction, while expressed *Fab-7* and *Mcp* PREs show their highest abundance in the earlier fractions (f1 and f2, respectively). In line with these findings, promoters of repressed genes are late replicating in both cell lines, while the region at the 5′end of *AbdB* gene, differentially expressed in the two cell lines, shows different replication timing, being mid replicating in S2 cells and early replicating in S3 (Figure 4D and Figure S5C). These data suggest that while in S2 cells the entire BX-C forms a single late replicating structural unit, in S3 cells the BX-C is divided into two structural domains showing distinct replication timing.

## Discussion

The epigenome in its overall complexity, including covalent modifications of DNA and histones, higher-order chromatin structures and nuclear positioning, influences transcription and replication programs of the cell. It is well known that timing of DNA replication is correlated with relative transcription state, in particular transcriptionally active genes tend to replicate early and inactive genes tend to replicate late (for a review, see [1], [45], [46]). However, in recent years, genome-wide analyses revealed several exceptions to this rule [18], [47], [48]. These and other evidence suggested that the transcriptional potential of chromatin, expressed as histone modifications and transcription factors binding (rather than the process of transcription *per se*) is most closely related to replication timing [18], [49], [50]. A recent work in *Drosophila* has shown that the selection and the timing of firing of replication origins are associated with distinct sets of chromatin marks and DNA binding proteins [34]. This reinforces previous works showing that mutation, overexpression, depletion or tethering of chromatin modifying proteins to specific loci in yeast, *Drosophila* and vertebrates determines changes in replication timing locally or/and at a global level [25], [51]–[56]. In mammals, it has been suggested that higher-order chromatin structures more than basal epigenome modifications better correlate with replication timing profiles [57], [58]. Although several proteins have been reported to control higher-order chromatin structure formation, their role in replicon structure and replication timing regulation remains to be elucidated. Among these, cohesins have been shown to co-localize with ORC binding sites [59], [60] and to influence replication origin choice and density through the regulation of specific chromatin loops [59]. Previously, we and others reported that PcG proteins are key regulators of higher-order chromatin structures and that condensins complex components and Topoisomerase II take part in PRE and BX-C silencing function [11]–[13], [41], [61]. Moreover, depletion of the mammalian PC homologue M33 determines a switch of the INK4a/ARF locus replication timing [62], suggesting a role for PcG proteins in the regulation of replication programs at their targets.

However, the interplay between PcG-mediated silencing, higher-order structures and control of replication timing in *Drosophila* has not been elucidated. We first addressed this issue on a genome-wide level finding that H3K27me3 enriched and PRC2 bound sequences replicate later than their unbound counterparts (Figure 1A, 1B). Surprisingly, the same is not true for PRC1 or PhoRC target sites, where the binding of PcG proteins does not significantly correlate with genome-wide replication timing distributions (Figure 1C–1E), highlighting a difference between PRC1, PhoRC and PRC2 complexes at a genome-wide scale. Notably, replication timing is more correlated to PRC1 binding at transcribed TSSs than at silenced TSSs (Figure 1D–1F).

To investigate the possible contribution of PRC1 and other PcG complexes at repressed genes, we decided to analyse in detail the functional interplay between PcG-dependent epigenetic signatures and maintenance of replication programs at one of the major PcG targets: the *Drosophila* BX-C. After depletion of single PcG proteins in S2 cells, we found reactivation of BX-C genes and their related PREs (Figure S2A). Interestingly, depletion of PHO protein causes only a mild effect on homeotic genes transcription, although this protein has been reported to be required for recruitment of PRC1 and 2 [6]. This suggests that multiple additional mechanisms of recruitment, such as ncRNAs or other protein partners, may act simultaneously at PcG target loci, as described particularly in mammalian cells (for a review, see [4]). Interestingly, also 3C analysis in single PcG depleted cells reveals a different response to E(z) depletion with respect to PC and PHO depletions. In particular, in PC and PHO depleted cells we could see no change or the small reduction of some PRE-PRE and PRE-promoter BX-C interactions, while in E(z) depleted cells even an increase in specific crosslinking frequencies for some interactions was observed (Figure 2). Moreover, both 3C and replication timing analysis in single PcG depleted cells (Figure 2) show that transcription *per se* cannot dramatically perturb the BX-C higher-order structures neither change the timing of replication. This result is in agreement with recent findings in mammalian cells showing that spatial chromatin organization and replication timing are not a direct consequence of transcription [50].

Conversely, simultaneous depletion of components of the three major PcG complexes (PhoRC, PRC2 and PRC1) determines major changes in BX-C transcription as well as in higher-order structure and an anticipation in replication timing (Figure 3; Figure S4C–S4E), suggesting that PcG proteins act synergistically on three-dimensional structures and on replication program maintenance. In line with these findings, in recovered cells, BX-C topological structure and PRE replication timing are indistinguishable from controls (Figure 3, Figure S4), suggesting that the observed variations are not sufficient to determine a stable epigenetic switch. In this context the more stable contacts might hamper an irreversible disruption of the three-dimensional BX-C structure (Figure S4F).

Our findings were further confirmed by the comparison of two different cell lines: S2 and S3 that differ for their embryonic origin. We have previously shown that in S3 cells, active transcription of *AbdB* is associated with different topological conformation of the locus, where *AbdB* gene and its regulative PREs are topologically separated from the other repressed and clustered epigenetic elements of the locus [13]. We found that distinct chromatin structures in S2 and S3 are associated with different replication timings (Figure 4, Figure S5), thus confirming that these epigenetic parameters vary in parallel.

Our analysis, in line with recent observations [50], [63], [64] indicate that the genome may be organized into distinct structural and functional domains in which DNA regions that stay together replicate together as a stable unit for many cell generations irrespective of single gene transcription state. It was shown that major adjustments of chromatin higher-order structure and replication program are necessary for a correct differentiation and are required for reprogramming of cell identity [65], [66]. The high stability of higher-order chromatin structures and replication programs can explain one of the underlying molecular basis counteracting cellular reprogramming and representing an epigenetic barrier [20] and PcG complexes may play an important role in the maintenance of this barrier. Our data show that correct levels of PcG components can fully restore silencing, higher-order structures and late replication timing at derepressed BX-C gene loci. Of course, we do not exclude that additional functions may be involved in the maintenance of these epigenetic parameters either at the BX-C and in the rest of the genome. For example, other factors involved in the regulation of higher-order chromatin structure, including the insulator CTCF protein, condensin complex subunits and Topoisomerase II, were shown to have a role in PcG-mediated gene silencing function [61], [67]. Interestingly, Topoisomerase II has been shown to be required for a global resetting of replicon organization in the context of somatic cell reprogramming [65]. Hence, a deeper understanding of the functional interplay between epigenetic mechanisms modulating the stability of higher-order chromatin structure and replication program will be crucial to unravel the molecular basis of maintenance of cell identity and its metastability in developmental and pathogenic processes.

## Materials and Methods

The following public available data sets in S2 cells were used for bioinformatic analyses (accession numbers are indicated in parenthesis). The genome-wide replication timing profile (GSM336376) was generated in [25] using Affymetrix tiling arrays. Pre-processed and normalized data were used for the analysis; E(z) (modENCODE_284), H3K27me3 (modENCODE_298) and PHO (modENCODE_3894). ChIP-chip profiles were downloaded from the modENCODE data warehouse as wiggle files containing smoothed M-values. PRC1 core components (PC, Psc and Ph) and Trx ChIP-Seq profiles as well as RNA-Seq gene expression profile were generated in [6] (GSE24521) and processed starting from fastq files.

### Definition of unique TSSs

Bioinformatic and statistical analyses have been performed using R (R Development Core Team, R: A Language and Environment for Statistical Computing, 2011, Vienna, Austria), Bioconductor and custom scripts. Ensembl gene annotations were pre-processed to obtain a set of unique Transcription Start Sites (TSSs, n = 9268). A TSS was defined as unique if no other TSS within a 2 kb window centered on it was annotated. The 1 kb window centered on a given unique TSS was used to define its promoter region. The replication timing of each promoter was computed as the median replication timing of the probes in the tiling array entirely mapping within the promoter region. Promoters with less than 10 mapping probesets were discarded (n = 67) in order to increase the robustness of replication timing estimates, rendering a set of 9201 unique promoters (simply referred to as promoters in the following) further considered for the analysis. Promoters were classified in transcriptional activity classes (0–4) according to the expression level of the corresponding genes. Non-transcribed promoters were assigned to class 0, whereas transcribed promoters were classified according to expression level quartiles (classes 1–4). Transcriptional classes 0 and 1 were considered as inactive promoters (OFF) whereas classes 2–4 defined active promoters (ON).

### Computing enrichments at promoters

PRC1 core components and Trx enrichments as well as RNA expression values at promoters were computed as described in [6]. H3K27me3, E(z) and PHO enrichments at promoters were estimated as the median smoothed M-value of probes entirely mapping within the promoters. Promoters significantly enriched for a given feature were defined using the following conservative approach based on the estimation of the genome-wide distribution of probe levels. First, a Gaussian Mixture Model (GMM) was fit to genome-wide smoothed M-values X. This is equivalent to say that the distribution of X was modelled as a mixture of two univariate Gaussian components
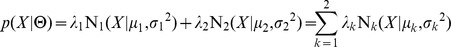
where 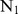 is the distribution of probe values in non-enriched regions and N_2_ is the distribution of probe values in enriched regions. Second, the parameters vector 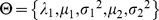 was estimated using a Maximum Likelihood approach via Expectation Maximization. Then, Bayesian inference was used to compute posterior probabilities for each individual probe. These can be viewed as the responsibility that component k takes in explaining the probe value. Each probe was then classified according to a Maximum a Posteriori (MAP) criterion, namely it was assigned to the class that maximizes the posterior probability. Finally, a promoter was called significantly enriched for a given feature if at least 80% of the probes mapping within the promoter region were assigned to class k = 2 meaning that overall probe levels are consistently more likely to originate from an enriched region. Notice that this choice leads to a rather conservative estimate of the number of significantly enriched promoters.

### Bootstrap and weighted bootstrap

Given a set of promoters, the percentile confidence interval for their mean replication timing was computed using nonparametric bootstrap [68]. A resampling depth of 10^4^ and a significance level 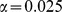 were used for all the analyses. The same parameters were applied for the weighted bootstrap with importance weights assigned to each promoter depending on its transcriptional activity class (ON/OFF) as inversely proportional to the cardinality of the class (i.e. the number of promoters belonging to the class).

### Cell cultures

*Drosophila* embryonic S2 cells were grown at 25°C in serum-free insect culture medium (HyQ SFX; Hyclone, Logan, UT) supplemented with penicillin/streptomycin. *Drosophila* embryonic S3 cells were grown at 25°C in Schneider's medium (Gibco#11720-034) supplemented with penicillin/streptomycin.

### RNAi

Exonic fragments of 600 bp, 1400 bp, 658 bp or 810 bp, respectively, from *Gfp*, *Pc*, *Pho* or *E(z)* genes, were amplified by PCR, creating T7 polymerase binding sites for the transcription of both strands. RNAi was performed as described previously [69]. Briefly, cells were diluted at 1*10^6^/ml and transfected with 2 micrograms of dsRNA. Three rounds of transfection were performed. Primer sequences used for PcG knock down: Gfp 5′ACGTAAACGGCCACAAGTTC3′-5′TGCTCAGGTAGTGGTTGTCG3′; Pc 5′ATTGGCAAGTTAAGCACGGGCA3′-5′ACATCCTGGATCGCCGCCTCA3′; Pho 5′ACAGTACGATGAAGATATAGGC3′-5′TGATCTGAACTGAGCTTATAGG3′; E(z) 5′TCGAAGGCATTATGAATAGCAC3′-5′ATCCGCATCTTCAGTCTCC3′.

### Replication timing analysis

Exponentially growing cells (1×10^6^ cells/ml) were cultured in presence of 50 µM Bromodeoxyuridine (BrdU) for 60 min. For sorting, cells were divided into aliquots containing 5×10^6^ cells per tube, washed with cold PBS, resuspended in 0.5 ml of cold PBS, fixed with drop-by-drop addition of 5 ml of 70% cold ethanol and incubated for 1 h on ice. Cells were then washed with PBS, resuspended in PBS/RNase A (1 mg/ml) 30 min at 37°C followed by addition of Propidium Iodide (20 µg/ml) and incubated 30 min in the dark at 4°C. On the basis of DNA content, cells were sorted into different S phase fractions using four selective gates. Equal numbers of cells from each cell cycle fraction (100,000) were sorted (using a Moflo, Coulter) into microcentrifuge tubes containing lysis buffer (50 mM TrisHCl pH 8; 10 mM EDTA; 0,8% SDS; supplemented with 0.2 mg of proteinase K per ml). The samples collected by FACS were processed for replication timing analysis as previously described [70]. The preparations were analyzed by real-time PCR. The relative abundance of locus-specific DNA in each cell-cycle fraction was calculated from the average values of threshold cycle (Ct), normalized to the Ct of a unique mitochondrial sequence as internal control, using the following equation: 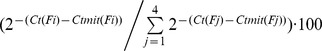, where i is one of the four fractions. All data points were generated from an average of six independent experiments. Primer sequences: EarlyCtrl-up 5′GGCGTGGCCTCATCGGATGG3′, EarlyCtrl-low 5′ACGAGTCCTGCCGCAAAGCC3′; LateCtrl-up 5′AAAGGCCTGGTTCGGCTGGC3′, LateCtrl-low 5′TTGCTACTTGCCGTGCGCGA3′;′ mitochondria up 5′AGCAACAGGATTCCACGGAATTC3′, mitochondria low 5′ATCATGCAGCTGCTTCAAAACCA3′; fab7-up 5′GAAAATGCCCAACAAAATGC3′, fab7-low 5′CGCTGTCTCGCCTCTTCTTC3′; mcp-up 5′TGCGGACGCCATTTGACAC3′, mcp-low 5′GAGCCACGCAGCGAGTTC3′: bxd-up 5′AGTTATCGGCACTTTGGTTCTG3′, bxd-low 5′GTAATTATCCAAACAAGCGACGG3′; bx-up 5′TTATTGTTGCTACACCGCTG3′, bx-low 5′AGTAGGTGCCGCGTATGTG3′; Ubxp-up 5′TCAGCCCTCCTCCATGATG3′, Ubxp-low 5′CCAAATCGCAGTTGCCAGTG3′; abdAp-up 5′TTGAGTCAGGGAGTGAGCC3′, abdAp-low 5′CGCTTTGAGTCGTTGGAGAC3′; AbdBpγ-up 5′TCGGAAGATTGTATTTGTGCGG3′, AbdBpγ-low 5′CAGTACGACAGTTCAGATGC3′; 5′UTRAbdBA-up 5′AGACAGCGGAGAACTCGCAC3′, 5′UTRAbdBA-low 5′TTG CCAATAGTCTG CAATTACAC3′.

### Chromosome conformation capture (3C)

The 3C assay was performed as previously described [13]. The 3C preparations were analyzed by real-time PCR. Primer sequences: int2-up 5′TTATCCACGGACGGCAGTC3′, int2-low 5′TCTGTGGGATTTGTGGGATC3′; AbdBpγ-up 5′ATAGATGGGCTGAGTGAGAG3′; Fab7-up 5′CTCACTTCTCCATGGCCTG3′; mcp22b-up 5′ATAGAAGTCAACATCCAGGC3′; mcp23-up 5′GGCCTGTCGAAGGAACGC3′; abdAp-up 5′ATGGCGCCAATGTGCTCTG3′; bxd-up 5′CCTTAGCACGTTGTCAAGTG3′; bx-up 5′AGTGATAATTGGTCCGGGAG3′.

### Real-time PCR analysis

Total RNA was isolated with Trizol reagent (Invitrogen). 1 µg of RNA from each sample was subjected to cDNA synthesis using a QuantiTect reverse transcription kit (Qiagen). DNA from BrdU immunoprecipitation, 3C or cDNA preparation was amplified in 20 µl reaction mixtures in the presence of 10 µl 2× QuantiTect SYBR Green master mix (Qiagen) and 0.5 µM of corresponding primers. Real-time PCR was performed using DNA Engine Opticon 2 (MJ) apparatus. Copy number was determined using the cross-point (Cp) value, which is automatically calculated using the Opticon Monitor 2 software (MJ). Primer sequences used for transcriptional analyses: RTgapdh-f 5′AAGGGAATCCTGGGCTACAC3′, RTgapdh-r 5′ACCGAACTCGTTGTCGTACC3′; RTpho-f 5′TCAGTTGGTTCACACCGGTG3′, RTpho-r 5′GAGGTATCTTCACTCTGGCTG3′; RTpc-f 5′TTCAAGACTCAAGTGCTGCC3′, RTpc-r 5′CCATGGGAAATAAGCAGGAG3′; RTez-f 5′CTGTGGCTGAGATCAACTCC3′, RTez-r 5′GACAGGTCTTGGTCAGCATG3′; RTUbx-f 5′AGTGTCAGCGGCGGCAAC3′, RTUbx-r 5′AGTCTGGTAGAAGTGAGCCCG3′; RTabdA-f 5′CAAATACAACGCAACCCGAGAC3′, RTabdA-r 5′AGCGATCGTGTTGCTGCTG3′; RTAbdBA-f 5′AATCTCCAGCAGCAGCAGC3′, RTAbdBA-r 5′ TGCCGTGTGCCGCTTGACCG3′.

### Recovery

In the recovery experiment, depleted cells were diluted once a week in fresh medium without the addition of new dsRNA, for 30 days (approximately 20 cell divisions).

### Protein extraction and Western blot analyses

Total proteins were prepared by resuspending 2×10^6^ S2 cells in extraction buffer (50 mM TrisHCl pH 7.6; 0.15 M NaCl; 5 mM EDTA; 16 Protease Inhibitors; 1% Triton X-100). Three pulses of 10 sec sonication at 30% amplitude were performed to allow dissociation of protein from chromatin and solubilization. Extracts were analysed by SDS-PAGE using an 8% gel (37.5∶1 Acryl/Bis Acrylamide).

### Antibodies

Antibodies against PHO and E(z) were kindly provided by J. Muller. Actin (Santa Cruz I-19 sc-1616).

## Supporting Information

Figure S1 Gene expression is not the only determinant of replication timing. (A) Weighted bootstrap distributions of mean replication timing for H3K27me3 significantly enriched promoters (orange) and non-enriched promoters (light blue). See Material and Methods for details. Percentile confidence intervals (α = 0.025) are indicated with dashed vertical lines. (B) Promoters have been binary classified according to PRC1 binding in PRC1 non-bound (−) and bound (+) promoters (represented in light blue and orange, respectively). Among PRC1 bound promoters, the fraction of promoters co-bound by Trx ((+) Trx+) is shown in gray.
(TIF)

Figure S2Depletion of single PcG subunits differentially affects BX-C transcription. (A) Log scale quantification by real time-PCR of transcript levels, relative to GAPDH, of the BX-C homeotic genes (left panel) and PRE transcripts (right panel) in cell treated with PC-dsRNA (pink) E(z)-dsRNA (purple), PHO-dsRNA (light blue) normalized to GFP dsRNA treated cells used as control, represented in blue. All data points were generated from an average of at least four independent experiments. Standard error of the mean is indicated. (B) Western blot of total protein extracts showing the amount of PcG proteins in cells treated with dsRNA against PC, E(z) and PHO mRNA or against GFP as control. Actin was used as a loading control. (C) Cell-cycle profile of *D. melanogaster* S2 control and depleted cells after BrdU pulse labelling and propidium iodide staining. Cells between the G1 and G2 peaks are in S phase. Gates indicate the sorted fractions: f1 represents the earliest and f4 the latest S-phase fraction. (D) Enrichment of BrdU labelled DNA in the four FACS sorted fractions as quantified by real time PCR with primers specific for the three homeotic gene promoters (*AbdBA*, *abdA* and *Ubx*). The relative abundance of locus-specific DNA in each cell-cycle fraction is calculated from the average values of threshold cycle (Ct), normalized to the Ct of a mitochondrial sequence as internal control (Ctmit), using the following equation: 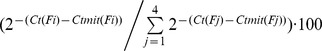, where *i* is one of the four fractions. (E) Replication timing of PREs and homeotic genes as measured by quantitative RT-PCR. Data obtained from GFP–dsRNA-treated cells are shown in blue while data obtained in cells treated with PC-dsRNA, E(z)-dsRNA or PHO-dsRNA are in pink, purple and light blue, respectively. Ratios between the amplified products in early and late S phase, using the following equation: 2^−(CtEarly-Ctmit^/2^−(CtLate-Ctmit)^ are shown. All data points were generated from an average of at least six independent experiments. Standard error of the mean is indicated. Two-tailed t-test was applied for statistical analysis. Asterisks indicate statistically relevant differences in comparison with early replicating control sequence: (α = 0.05). P values: GFP–dsRNA-treated cells: Early ctrl/Late ctrl: P = 0,06×10^−1^; Early ctrl*/Fab-7*: P = 0,06×10^−1^; Early ctrl*/Mcp*: P = 0,06×10^−1^; Early ctrl*/bxd*: P = 0,07×10^−1^; Early ctrl*/bx*: P = 0,06×10^−1^; Early ctrl*/abdAp*: P = 0,07×10^−1^; Early ctrl*/Ubxp*: P = 0,06×10^−1^. PC-dsRNA-treated cells: Early ctrl*/Fab-7*: P = 0,01; Early ctrl*/Mcp*: P = 0,01; Early ctrl*/bxd*: P = 0,02; Early ctrl*/bx*: P = 0,01; Early ctrl*/abdAp*: P = 0,02; Early ctrl*/Ubxp*: P = 0,02. E(z)-dsRNA-treated cells: Early ctrl*/Fab-7*: P = 0,02×10^−2^; Early ctrl*/Mcp*: P = 0,02×10^−2^; Early ctrl*/bxd*: P = 0,02×10^−2^; Early ctrl*/bx*: P = 0,02×10^−2^; Early ctrl*/abdAp*: P = 0,02×10^−2^; Early ctrl*/Ubxp*: P = 0,02×10^−2^. PHO-dsRNA-treated cells: Early ctrl*/Fab-7*: P = 0,01×10^−2^; Early ctrl*/Mcp*: P = 0,01×10^−2^; Early ctrl*/bxd*: P = 0,01×10^−2^; Early ctrl*/bx*: P = 0,02×10^−2^; Early ctrl*/AbdB5′UTR*: P = 0,02×10^−1^; Early ctrl*/abdAp*: P = 0,02×10^−2^; Early ctrl*/Ubxp*: P = 0,02×10^−2^.(TIF)

Figure S3 Replication timing profile of chromosome 3R in S2 cells at 1 kb resolution (light blue) obtained from GSM336376 and represented in logarithmic scale [21] (see Material and Methods). Loess smoothed signal is shown in dark blue (100 kb span). The genomic position of the BX-C is enclosed in the black rectangle. Its expansion details the Replication timing and H3K27me3 enrichment profiles at datasets nominal scale in the BX-C (gray-black and orange-blue tracks, respectively). Flybase protein-coding genes (light blue), PREs (dark blue) and primer sets targeting the *Abd-B* 5′UTR (red) are shown at the bottom. Red arrow indicates the *Abd-B* promoter replication timing.
(TIF)

Figure S4 Multiple PcG depletion causes substantial homeotic genes and PRE transcripts derepression and changes in replication timing. Data obtained in control cells are shown in blue, data obtained in cells treated with dsRNA against PC, E(z) and PHO and in recovered cells are in orange and violet, respectively. (A) Quantification by real time-PCR of transcript levels, normalized to GAPDH, of PcG mRNA. (B) Western blot of total protein extract showing the amount of PcG proteins in control cells, in cells depleted for PC, E(z) and PHO mRNA (left panels) and in recovered cells (right panels). Actin was used as a loading control. (C, D) Quantification by real time-PCR of transcript levels, normalized to GAPDH, of BX-C homeotic genes (C) and PRE transcripts (D). Data were generated from an average of at least five independent biological replicates. Standard error of the mean is indicated. (E) Replication timing of PREs and homeotic genes as measured by quantitative RT-PCR. Ratios between the amplified products in early and late S phase, using the following equation: 2^−(CtEarly-Ctmit)^/2^−(CtLate-Ctmit)^ are shown. All data points were generated from an average of at least four independent experiments. Standard error of the mean is indicated. Two-tailed t-test was applied for statistical analysis. Asterisks indicate statistically relevant differences between PREs and early and late replicating control sequences (α = 0.05). P values (comparison with late replicating control): Late Ctrl/*Fab7* P = 0,07×10∧^−1^; Late Ctrl/*Mcp* P = 0,03×10∧^−2^; Late Ctrl/*bxd* P = 0,02×10∧^−1^; Late Ctrl/*Ubxp* P = 0,06×10∧^−1^. P values (comparison with early replicating control): Early Ctrl/*Fab7* P = 0,04×10∧^−3^; Early Ctrl/*Mcp* P = 0,07×10∧^−3^; Early Ctrl/*bxd* P = 0,05×10∧^−3^; Early Ctrl/*Ubxp* P = 0,06×10∧^−2^. (F) Crosslinking frequency, normalized to the internal control, between two homeotic gene promoters (*AbdBγ* and *abdA*) and their functional PREs (*Fab-7* and *Mcp*, respectively) and between *Fab7*/*Mcp* and *bxd*/*bx* PREs. (G) Cell count of GFP–dsRNA-treated cells (blue) and PC, E(z), PHO dsRNA treated cells (orange) after each one of the three rounds of transfection. Standard error of the mean is indicated. (H) Enrichment of BrdU labelled DNA in the four FACS sorted fractions as quantified by real time PCR with primers specific for PREs and controls in GFP-dsRNA treated cells after recovery.
(TIF)

Figure S5 Different epigenetic signatures and replication timing profiles in S2 and S3 cell lines. (A) Log scale quantification by real time-PCR of transcript levels, normalized to GAPDH, of the BX-C homeotic genes (left panel) and PRE transcripts (right panel) in S3 cells (violet) compared to S2 (blue). All data points were generated from an average of at least three independent experiments. Standard error of the mean is indicated. (B) In S2 cells, the BX-C locus adopts a condensed structure in which all the Polycomb group (PcG)-bound elements are interacting together. In S3 cells, the PRE–promoter interaction, in the *AbdB* domain, is lost, whereas the rest of the BX-C retains its clustered conformation. (C) Replication timing of PRE as measured by quantitative RT-PCR in S2 cells (blue) and in S3 cells (violet). Ratios between the amplified products in early and late S phase, using the following equation: 2^−(CtEarly-Ctmit)^/2^−(CtLate-Ctmit)^ are shown. We amplified positive controls for the early and late S phase. Asterisks indicate statistically relevant differences in comparison with early replicating control sequence; α = 0.05. P values: S2: *Early ctrl/Late ctrl*: P = 0,05×10^−2^; *Early ctrl/Fab7* P = 0,05×10^−2^; *Early ctrl/Mcp* P = 0,05×10^−2^; *Early ctrl/bxd* P = 0,05×10^−2^; *Early ctrl/bx* P = 0,05×10^−2^; *Early ctrl/abdAp* P = 0,05×10^−2^; *Early ctrl/Ubxp* P = 0,05×10^−2^. S3: *Early ctrl/Late ctrl*: P = 0,01×10^−2^; *Early ctrl/bxd* P = 0,06×10^−3^; *Early ctrl/bx* P = 0,06×10^−3^; *Early ctrl/abdAp* P = 0,03; *Early ctrl/Ubxp* P = 0,02.
(TIF)
