# Supplementary figures and images for: PcG-Mediated Higher-Order Chromatin Structures Modulate Replication Programs at the Drosophila BX-C
Source: PLoS Genet. 2013 Feb 21;9(2):e1003283. doi: 10.1371/journal.pgen.1003283 (PMC3578750; doi:10.1371/journal.pgen.1003283)

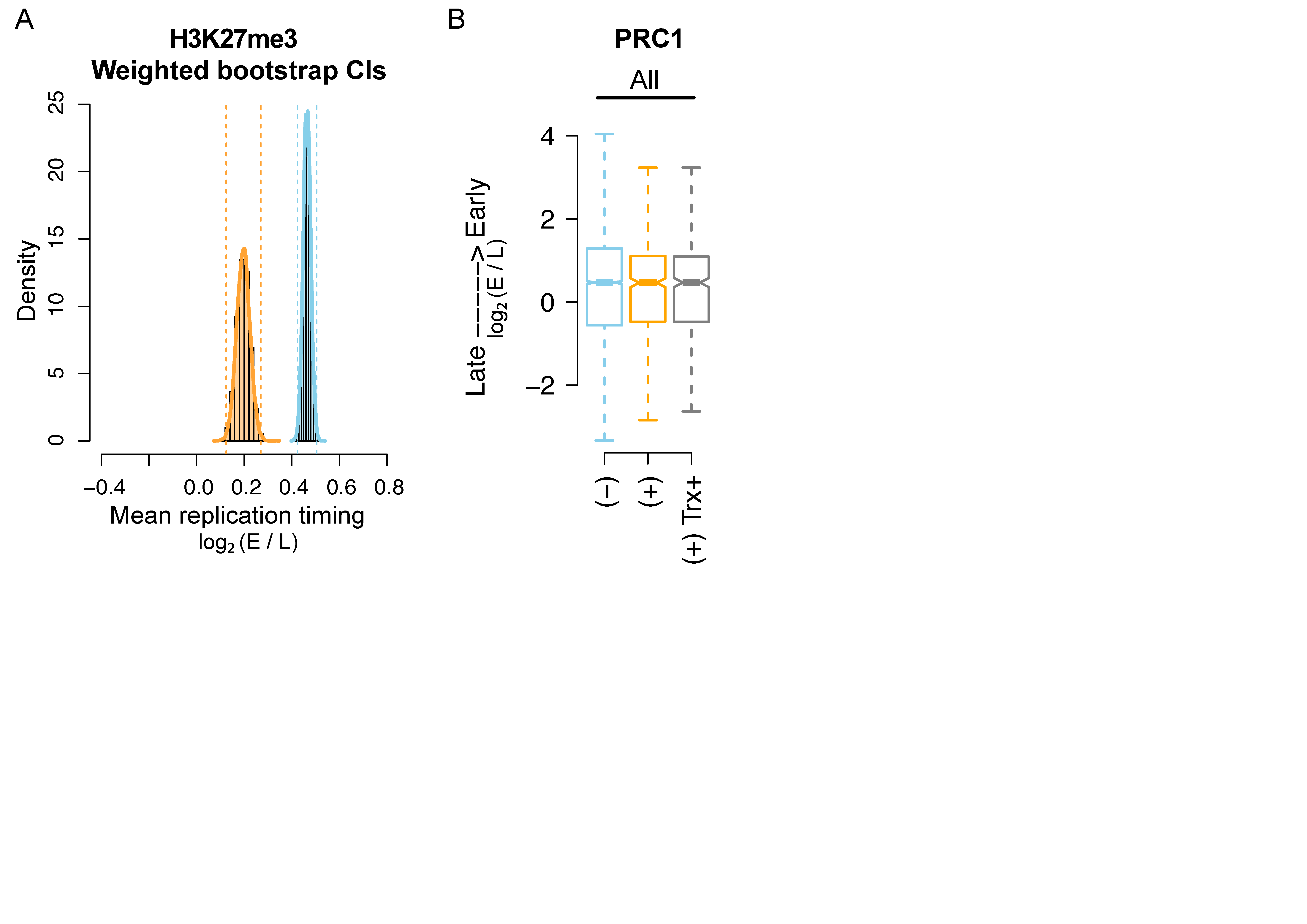

Supplement: Figure S1 — Gene expression is not the only determinant of replication timing. (A) Weighted bootstrap distributions of mean replication timing for H3K27me3 significantly enriched promoters (orange) and non-enriched promoters (light blue). See Material and Methods for details. Percentile confidence intervals (α = 0.025) are indicated with dashed vertical lines. (B) Promoters have been binary classified according to PRC1 binding in PRC1 non-bound (−) and bound (+) promoters (represented in light blue and orange, respectively). Among PRC1 bound promoters, the fraction of promoters co-bound by Trx ((+) Trx+) is shown in gray. (TIF) [file pgen.1003283.s001.tif]

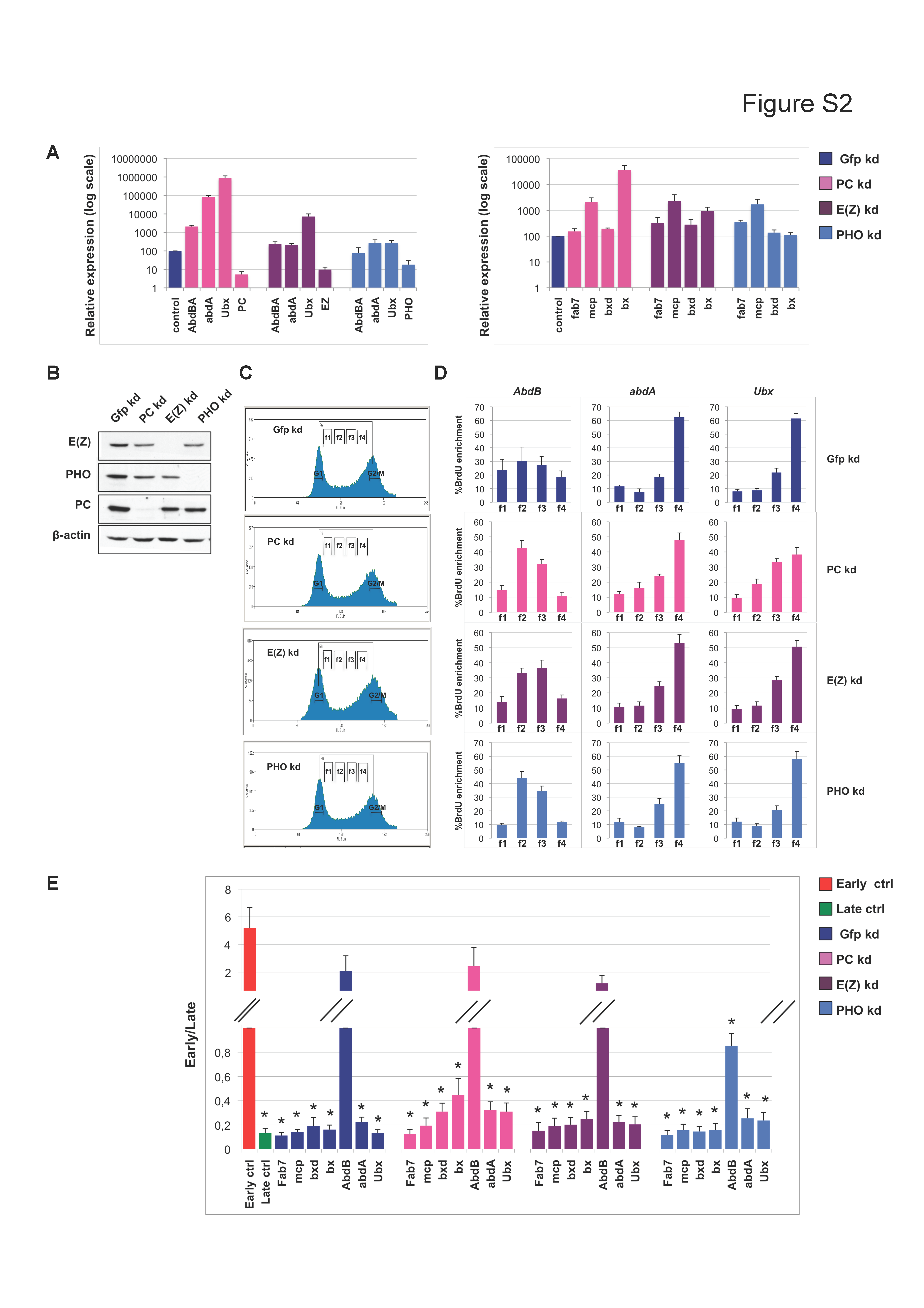

Supplement: Figure S2 — Depletion of single PcG subunits differentially affects BX-C transcription. (A) Log scale quantification by real time-PCR of transcript levels, relative to GAPDH, of the BX-C homeotic genes (left panel) and PRE transcripts (right panel) in cell treated with PC-dsRNA (pink) E(z)-dsRNA (purple), PHO-dsRNA (light blue) normalized to GFP dsRNA treated cells used as control, represented in blue. All data points were generated from an average of at least four independent experiments. Standard error of the mean is indicated. (B) Western blot of total protein extracts showing the amount of PcG proteins in cells treated with dsRNA against PC, E(z) and PHO mRNA or against GFP as control. Actin was used as a loading control. (C) Cell-cycle profile of D. melanogaster S2 control and depleted cells after BrdU pulse labelling and propidium iodide staining. Cells between the G1 and G2 peaks are in S phase. Gates indicate the sorted fractions: f1 represents the earliest and f4 the latest S-phase fraction. (D) Enrichment of BrdU labelled DNA in the four FACS sorted fractions as quantified by real time PCR with primers specific for the three homeotic gene promoters (AbdBA, abdA and Ubx). The relative abundance of locus-specific DNA in each cell-cycle fraction is calculated from the average values of threshold cycle (Ct), normalized to the Ct of a mitochondrial sequence as internal control (Ctmit), using the following equation: , where i is one of the four fractions. (E) Replication timing of PREs and homeotic genes as measured by quantitative RT-PCR. Data obtained from GFP–dsRNA-treated cells are shown in blue while data obtained in cells treated with PC-dsRNA, E(z)-dsRNA or PHO-dsRNA are in pink, purple and light blue, respectively. Ratios between the amplified products in early and late S phase, using the following equation: 2−(CtEarly-Ctmit/2−(CtLate-Ctmit) are shown. All data points were generated from an average of at least six independent experiments. Standard error of the mean [file pgen.1003283.s002.tif]

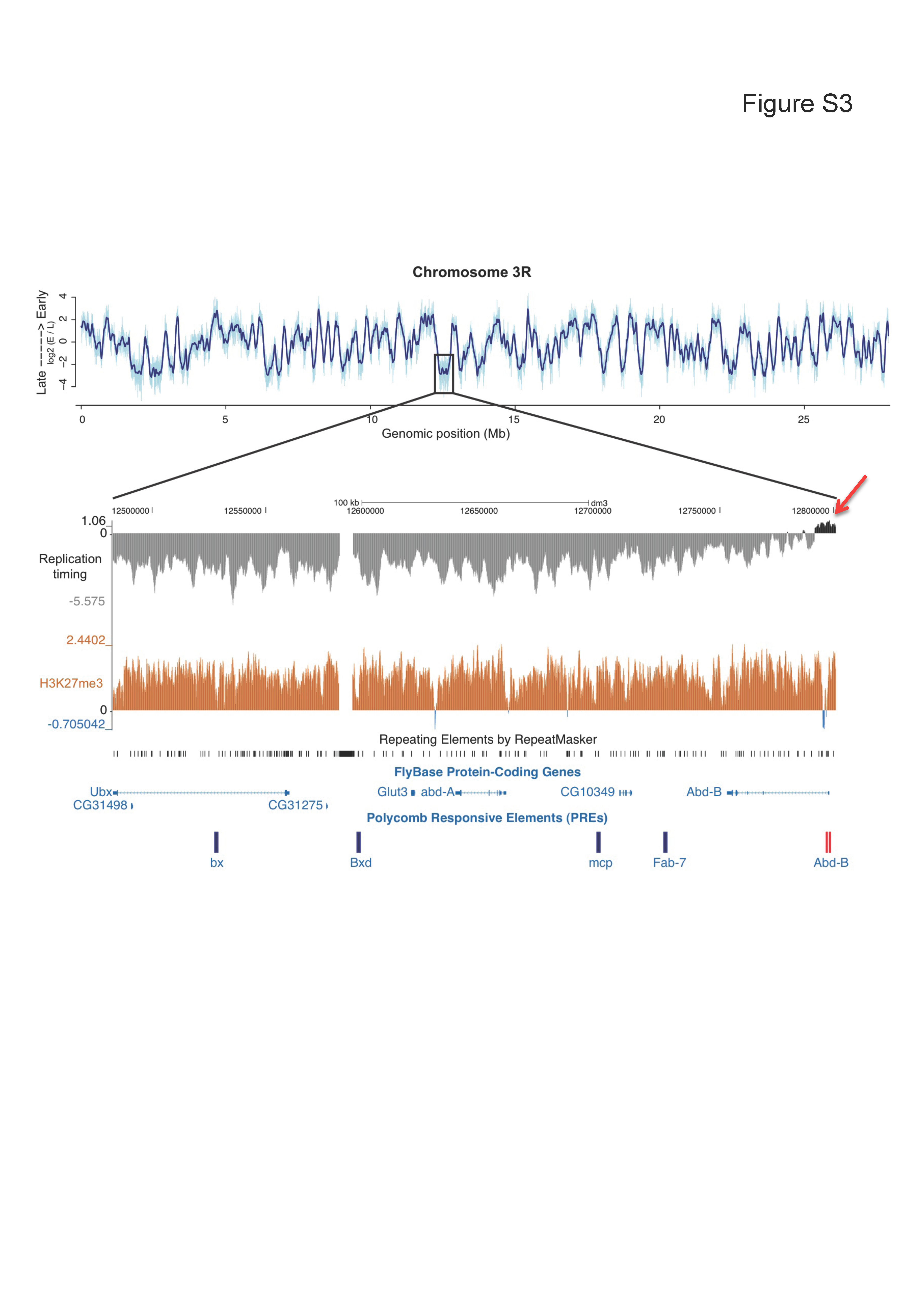

Supplement: Figure S3 — Replication timing profile of chromosome 3R in S2 cells at 1 kb resolution (light blue) obtained from GSM336376 and represented in logarithmic scale [21] (see Material and Methods). Loess smoothed signal is shown in dark blue (100 kb span). The genomic position of the BX-C is enclosed in the black rectangle. Its expansion details the Replication timing and H3K27me3 enrichment profiles at datasets nominal scale in the BX-C (gray-black and orange-blue tracks, respectively). Flybase protein-coding genes (light blue), PREs (dark blue) and primer sets targeting the Abd-B 5′UTR (red) are shown at the bottom. Red arrow indicates the Abd-B promoter replication timing. (TIF) [file pgen.1003283.s003.tif]

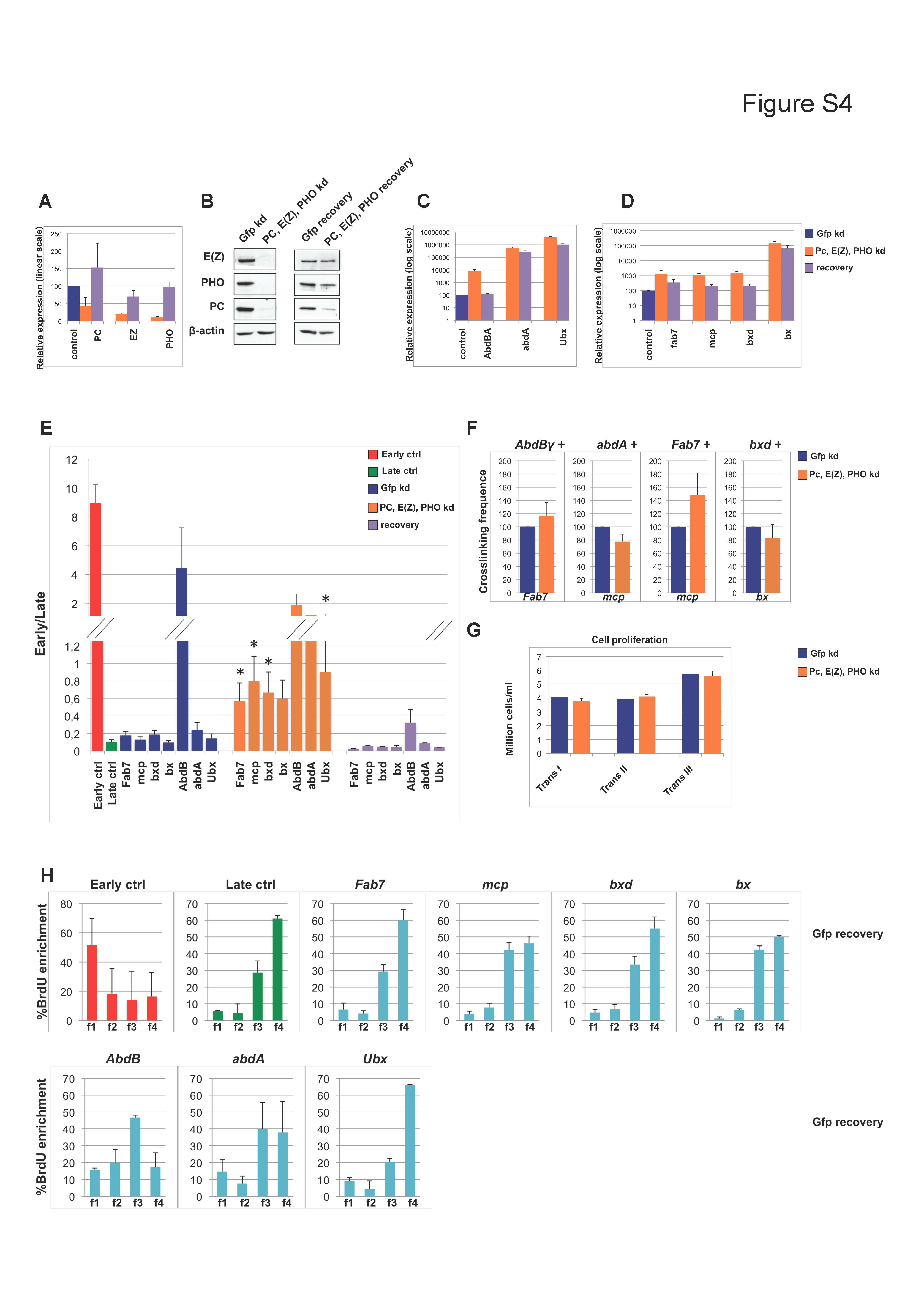

Supplement: Figure S4 — Multiple PcG depletion causes substantial homeotic genes and PRE transcripts derepression and changes in replication timing. Data obtained in control cells are shown in blue, data obtained in cells treated with dsRNA against PC, E(z) and PHO and in recovered cells are in orange and violet, respectively. (A) Quantification by real time-PCR of transcript levels, normalized to GAPDH, of PcG mRNA. (B) Western blot of total protein extract showing the amount of PcG proteins in control cells, in cells depleted for PC, E(z) and PHO mRNA (left panels) and in recovered cells (right panels). Actin was used as a loading control. (C, D) Quantification by real time-PCR of transcript levels, normalized to GAPDH, of BX-C homeotic genes (C) and PRE transcripts (D). Data were generated from an average of at least five independent biological replicates. Standard error of the mean is indicated. (E) Replication timing of PREs and homeotic genes as measured by quantitative RT-PCR. Ratios between the amplified products in early and late S phase, using the following equation: 2−(CtEarly-Ctmit)/2−(CtLate-Ctmit) are shown. All data points were generated from an average of at least four independent experiments. Standard error of the mean is indicated. Two-tailed t-test was applied for statistical analysis. Asterisks indicate statistically relevant differences between PREs and early and late replicating control sequences (α = 0.05). P values (comparison with late replicating control): Late Ctrl/Fab7 P = 0,07×10∧−1; Late Ctrl/Mcp P = 0,03×10∧−2; Late Ctrl/bxd P = 0,02×10∧−1; Late Ctrl/Ubxp P = 0,06×10∧−1. P values (comparison with early replicating control): Early Ctrl/Fab7 P = 0,04×10∧−3; Early Ctrl/Mcp P = 0,07×10∧−3; Early Ctrl/bxd P = 0,05×10∧−3; Early Ctrl/Ubxp P = 0,06×10∧−2. (F) Crosslinking frequency, normalized to the internal control, between two homeotic gene promoters (AbdBγ and abdA) and their functional PREs (Fab-7 and Mcp, respectively) and between Fab7/Mcp and bxd/bx PREs. (G) [file pgen.1003283.s004.tif]

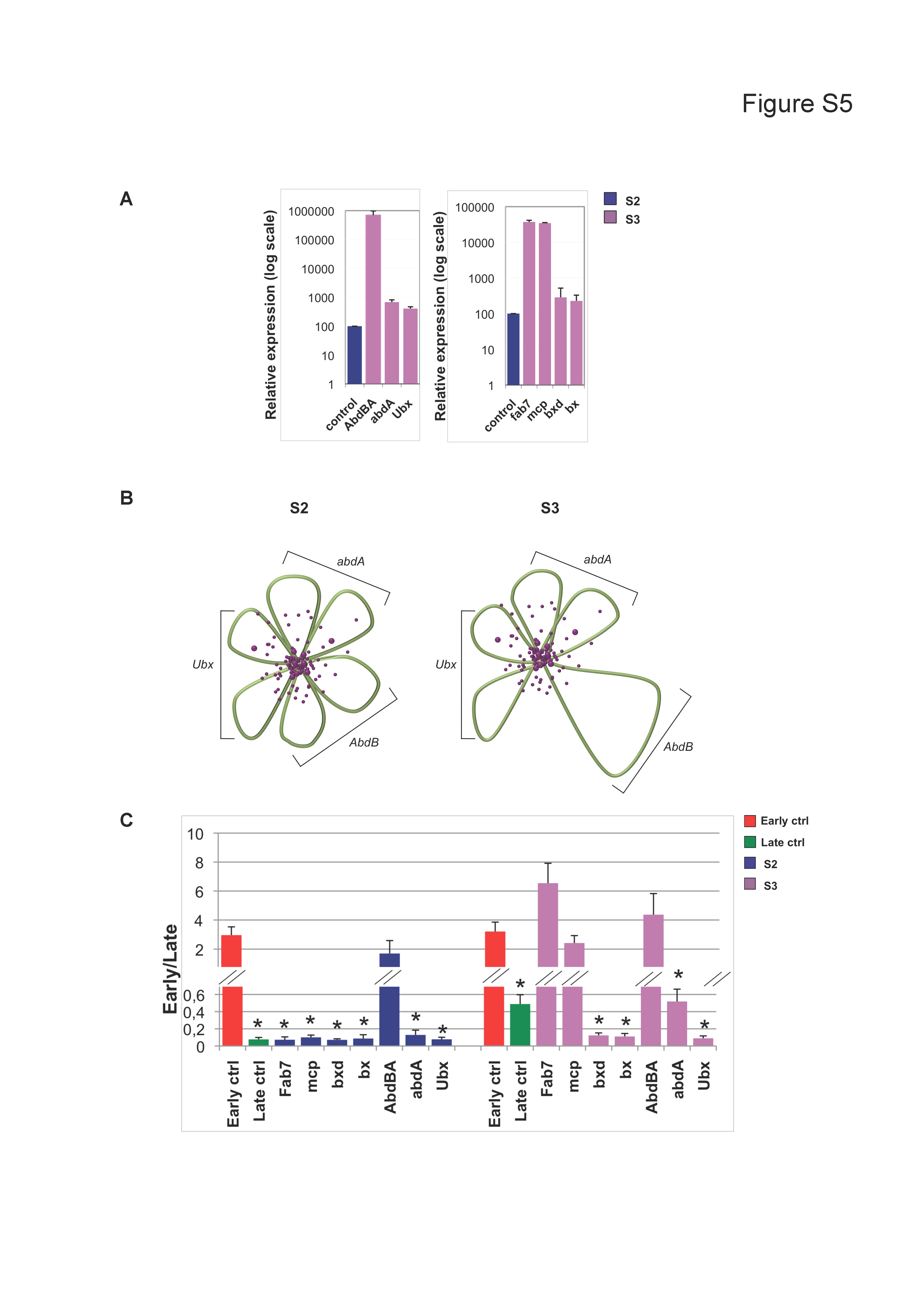

Supplement: Figure S5 — Different epigenetic signatures and replication timing profiles in S2 and S3 cell lines. (A) Log scale quantification by real time-PCR of transcript levels, normalized to GAPDH, of the BX-C homeotic genes (left panel) and PRE transcripts (right panel) in S3 cells (violet) compared to S2 (blue). All data points were generated from an average of at least three independent experiments. Standard error of the mean is indicated. (B) In S2 cells, the BX-C locus adopts a condensed structure in which all the Polycomb group (PcG)-bound elements are interacting together. In S3 cells, the PRE–promoter interaction, in the AbdB domain, is lost, whereas the rest of the BX-C retains its clustered conformation. (C) Replication timing of PRE as measured by quantitative RT-PCR in S2 cells (blue) and in S3 cells (violet). Ratios between the amplified products in early and late S phase, using the following equation: 2−(CtEarly-Ctmit)/2−(CtLate-Ctmit) are shown. We amplified positive controls for the early and late S phase. Asterisks indicate statistically relevant differences in comparison with early replicating control sequence; α = 0.05. P values: S2: Early ctrl/Late ctrl: P = 0,05×10^−2^; Early ctrl/Fab7 P = 0,05×10^−2^; Early ctrl/Mcp P = 0,05×10^−2^; Early ctrl/bxd P = 0,05×10^−2^; Early ctrl/bx P = 0,05×10^−2^; Early ctrl/abdAp P = 0,05×10^−2^; Early ctrl/Ubxp P = 0,05×10^−2^. S3: Early ctrl/Late ctrl: P = 0,01×10^−2^; Early ctrl/bxd P = 0,06×10^−3^; Early ctrl/bx P = 0,06×10^−3^; Early ctrl/abdAp P = 0,03; Early ctrl/Ubxp P = 0,02. (TIF) [file pgen.1003283.s005.tif]
